# Tissue and Stem Cell Sourced Extracellular Vesicle Communications with Microglia

**DOI:** 10.1007/s12015-020-10011-y

**Published:** 2020-07-25

**Authors:** Samantha E. Spellicy, Steven L. Stice

**Affiliations:** 1 Regenerative Bioscience Center, Department of Animal and Dairy Science, Rhodes Center for Animal and Dairy Science, University of Georgia, 425 River Road, Athens, GA 30602 USA; 2 University System of Georgia MD/PhD Program, Medical College of Georgia, Augusta, GA 30912 USA; 3 Aruna Bio Inc, Athens, GA 30602 USA

**Keywords:** Extracellular vesicles, Exosomes, Microglia, Inflammation, Stem cells

## Abstract

Extracellular vesicles (EVs), nano- to micro- sized vesicles released from cells, have garnered attention in recent years for their role in intercellular communication. Specifically, EVs from various cell sources including stem cells, have shown to have an exacerbatory or therapeutic effect in the content of pro- and anti-inflammatory environments through their interaction with immune recipient cells. This review aims to the coalescence information surrounding EVs derived from various sources and their interaction with microglia in neutral, anti, and pro- inflammatory environments. Overall, in homeostatic environments, EVs from many CNS lineages have been shown to have specific interactions with recipient microglia. In complex inflammatory environments, such as the tumor micro-environment (TME), EVs have been shown to further influence immune dampening through transition of microglia to a more M2-like phenotype. While not advantageous in the TME, this effect can be harnessed therapeutically in proinflammatory neurological conditions such as stroke, Alzheimer’s, and Parkinson’s. EVs derived from various stem cell and non-stem cell derived sources were found to attenuate proinflammatory responses in microglia in in vitro and in vivo models of these conditions. EVs loaded with anti-inflammatory therapeutics furthered this anti-inflammatory effect on recipient microglia.

**Graphical Abstract:**
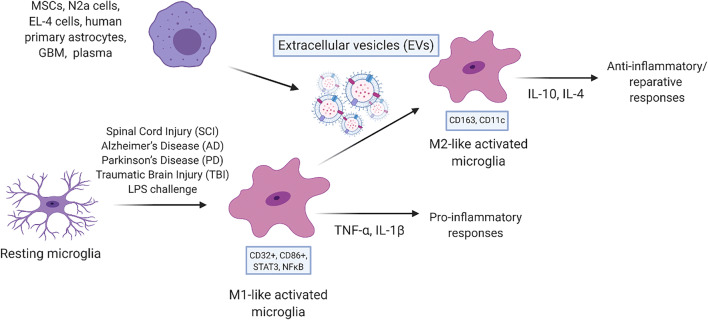
Extracellular Vesicles (EVs) from multiple cells types modulate microglial polarization. Cartoon depicting common ways microglia are activated through inflammatory and disease processes. EVs, derived from stem and non-stem sources, have been shown to attenuate proinflammatory responses in in vitro and in vivo.

Extracellular Vesicles (EVs) from multiple cells types modulate microglial polarization. Cartoon depicting common ways microglia are activated through inflammatory and disease processes. EVs, derived from stem and non-stem sources, have been shown to attenuate proinflammatory responses in in vitro and in vivo.

## Background

Extracellular vesicles (EVs), including exosomes and microvesicles, have garnered attention in recent years as a novel type of non-contact-mediated intercellular communication. These vesicles, of endosomal or plasma membrane origin, range in size from ~30 nm-1uM and are released extracellularly by all cell types to be taken up by various populations of recipient cells [1–4]. These vesicles contain a variety of proteins, mRNA, miRNA, and lipids derived from their parent cells [5]. While some groups have looked to characterize surface molecule expression and content for potential biomarker applications [6], the functional downstream effects of these EVs on recipient cells is also of scientific interest [7]. For example, mRNA transcripts found within EVs have been shown to be translated into functional proteins in recipient cells [5]. Furthermore, certain cell types, such as primary T lymphocytes, have been shown to selectively sort miRNAs into EVs with the help of RNA-binding proteins [8].

Common technical limitations of EV studies have made it difficult to monitor and investigate dynamic endogenous EV activity and obtain high purity EV RNA. Due to their small size, EV isolation methods (ultracentrifugation, ultrafiltration, immunoprecipitation, density gradient flotation, etc. [9] can affect the quantity and quality of isolated EVs and the EV-RNA [10, 11]. Some of these technical limitations can be mitigated through advancements in imaging, which can eliminate the need for EV isolation and allow direct observation of EV effects on recipient cell function, morphology, and activation. For example, 2-photon microscopy, allows for direct observation of endogenous EV production and uptake in CNS cell populations. Similar non-invasive imaging advancements also potentially surmount limitations associated with EV dosing paradigms, elucidating the collective effects of EVs on recipient cells without the need for direct analysis of EV cargo. Future studies should consider looking closely at EV cargo, such as EV RNA, to inform possible mechanisms of action for anti-inflammatory microglia responses noted in this review.

### Therapeutic Potential of EVs in Proinflammatory Conditions

Central nervous system (CNS)-derived EVs have shown great therapeutic potential. Neural stem cell-derived (NSC) EVs have significantly improved recovery in large and small animal models of ischemic stroke [12–14], as well as in models of traumatic brain injury (TBI) [15]. The parent cells of these EVs, neural stem cells, as well as neurosphere cells (NC) modulated the inflammatory microenvironment after stroke [16]. Some of the NSC EV therapeutic potential could be attributed to modulation of the neuroinflammatory environment. Since microglia are the resident immune cells of the CNS, the effect of EVs on microglia may play a major role in the efficacy of EVs in CNS injury.

### Characteristics of Microglial Polarization and Activation

The relationship between EVs and microglia is unique, not only in disease states, but also developmentally. Microglia finely regulate inflammation states in the brain and spinal cord. Environmental cues can shift resident microglia towards either an M1- or M2- like polarization [17, 18]. While, microglia actually embody a spectrum of polarization and do not explicitly fall into binary categorizations [19], these designations have traditionally been used as a general indication of microglia functional state. Here “M1-like” or “M2-like” is used in accordance with the cited study findings as a reference to which portion of this spectrum the microglia are predominantly embodying. M1-like microglia, expressing CD86^+^, CD206-, and CD16/32^+^ [20–22], produce cytokines such as INF-y, IL-6, TNF-a, IL1-B, KC/GRO/CINC. During activation, microglia also upregulate expression. M1-like microglia, expressing CD86^+^, CD206-, and CD16/32^+^ [20–22], produce cytokines such as INF-y, IL-6, TNF-a, IL1-B, KC/GRO/CINC. During activation, microglia also upregulate expression of ionizing calcium adaptor binding protein (IBA1), a commonly used marker for microglia. In turn, this polarization heightens the local proinflammatory environment. In development, M1-associated cytokines, such as TNF-a, Il-6, and IL-1, have been shown to be important factors contributing to synaptic plasticity and memory [23]. Conversely, M2-like microglia (CD86-, CD206^+^) [20] have increased gene expression of Arg-1, IL-10, and STAT6, and release CXCL1, GROα, neutrophil activating protein alpha, cytokine-induced neutrophil chemoattractant (CINC) inducing a more restorative local environment [24]. The studies discussed in this review utilize these polarization-associated changes in expression, transcription, and cytokine secretion to assess the effects of various EV populations on recipient microglia.

Functional transitions of microglia are accompanied by a change in microglia morphology. Upon activation, microglia undergo transitions from a more ramified, complex, aster-like morphology, to a more amoeboid, rounded, and swollen morphology [25, 26]. While morphological changes of microglia may be a less direct way to determine M1- or M2-like polarization of microglia than cytokine analysis, they do serve as a high-throughput way to gauge the inflammation status of microglia in vitro and in vivo [27]. Therefore, if EVs from various cell types are able to exert a specific, measurable effect on microglia polarization, evidenced through cytokine production or morphology, this anti-inflammatory property can be utilized therapeutically [28]. In acute proinflammatory neurological conditions such as stroke [29], TBI [30], spinal cord injury (SCI) [31], or chronic inflammatory conditions such as Parkinson’s [32] and Alzheimer’s [33], exogenous EVs can be provided to shift microglial polarization to more reparative or inactivated state.

Microglia not only play an essential role in tuning local inflammatory environments in the neuronal space, but they also have widespread holistic effects, serving as the major antigen-presenting cells in the brain parenchyma. For example, microglia have been shown to directly affect circulating peripheral T cell activation following injury and disease [34, 35]. Therefore, exogenously administered EVs could invoke changes in microglia activation in homeostatic or in neuroinflammatory injury and disease states which would not only have neuronal effects, but also systemic peripheral ramifications as well.

In contrast to recent reviews which have focused on the functional effects of microglia-derived EVs in various inflammatory states [36, 37], this review aims to coalesce information specifically surrounding the relationship between EVs derived from various cell sources, including stem cells and their effects on recipient microglia. Microglia-derived EVs are only considered in the context of their effects on recipient microglia. Here, the specificity of this relationship in neutral, pro- and anti-inflammatory conditions is examined, as well as the downstream functional effect on microglia polarization and activation.

## Regulation of Microglia by EVs

### The Role of EVs in Microglia Homeostasis and Development

#### *Interaction of EVs and microglia* In Vitro

In homeostatic states, EVs from multiple cell types have been shown to have a specific association with microglia. The extent and components of this specific association have been assessed for oligodendrocyte- [38], neuronal- [39], and astrocyte- [40] derived EVs on various in vitro microglia cell lines. Under homeostatic conditions, mouse oligodendroglia cell (Oli-Neu) EVs were taken up by primary microglia cultures [38, 41]. To test the specificity of this uptake, Oli-Neu EVs were applied to mixed primary brain cultures and their uptake by different recipient cells was compared. This study showed there was a specific uptake of Oli-Neu EVs by primary microglia not replicated by neurons, astrocytes, or oligodendrocytes [38].The degree of uptake of these EVs was decreased in the presence of phosphatidyl serine (PS) and phosphatidyl choline (PC, to a lesser extent)-containing liposome, or following inhibition with Dynasore, an inhibitor of dynamin-dependent endocytosis [38]. The dependence of PS epitope availability for efficient EV uptake by microglia has been observed in other in vitro models, as well. For example, blocking of the PS epitope on N2a (a neuroblastoma cell line)-derived EVs was shown to affect their uptake into BV2 cells (an immortalized microglia cell line) and primary microglia. Additionally, specific uptake of N2a EVs into BV2s and primary microglia was documented, but not into primary neurons [39]. Lastly, astrocyte-derived EVs (ADEVs) were shown to be taken up into the cytoplasm of primary microglia and then trafficked and colocalized to microglial endosomes [40]. These in vitro studies reinforce the notion that there is a robust, specific, and significant uptake of oligodendrocyte, neuronal, and astrocyte EVs by microglia in non-diseased or homeostatic conditions compared to other recipient cell types.

#### *Functional effects of EVs and microglia* In Vitro

In addition to cytokine release and surveillance, microglia are known to carry out a variety of other functions in non-diseased states such as neurite pruning. The effect of EVs on these other microglia process has also been assessed. Following exposure to differentiated and excited PC12 (a pheochromocytoma-derived mix of neuroblastic and eosinophilic cells) EVs, MG6 cells (a mouse microglial cell line) had an enhanced ability to remove degenerating neurites from PC12 cells compared to control non-exposed MG6 cells. This enhanced pruning effect was specific to PC12-EV engulfment, since uptake of NIH3T3/TIM4 EVs (TIM4-expressing mouse embryonic fibroblast line) did not produce the same pruning effect. PC12 EV uptake did not affect other classically understood microglia functions, such as phagocytosis of *E. coli*. The enhanced synaptic pruning response of MG6 microglia was due to a specific, detectable, and inhibitable upregulation of complement component 3 (C3) [42]. In contrast, astrocyte-derived extracellular vesicles (ADEVs) exposed to morphine increased activation of toll like receptor 7 (TLR7), ultimately impairing microglial phagocytosis processes [40]. This shows that while uptake of some EVs, such as PC12 derived EVs, may not affect phagocytic microglia processes, previous exposure of EVs to opioids or other conditions could affect these processes. Decreased phagocytosis was only reported in morphine-exposed ADEVs, thus further examination is required.

#### *Interaction of EVs and microglia* In Vivo

Specific EV and microglial association under homeostatic conditions has also been assessed in vivo*.* PKH26-labeled Oli-Neu EVs were injected intrathecally into C3CR1 (microglia)-EGFP transgenic mice, and 2-photon microscopy revealed 50% of EGFP-labeled microglia colocalized with PKH26-labeled Oli-Neu EVs within 400uM of the injection site [38]. This showed that even in the complex in vivo environment, a majority of microglia surrounding the injection site had successfully interacted with and taken up labeled Oli-Neu EVs. Experiments with other EV types reported similar results regarding EV uptake and clearance in developmental conditions [43]. Constitutively released CD9-GFP-positive subventricular zone (SVZ) NSC EVs were cleared in neonatal mice concurrently with an influx of IBA1-positive microglia. Furthermore, DiI-labeled SVZ NSC EVs revealed a pattern of uptake into IBA1^+^ and CD11b^+^ Cd68^+^ microglia This uptake was not effected by UV irradiation of nucleic acid EV content [43]. Upon investigation of N2a EV uptake, DiI-labeled EV were observed to colocalize with 93% of IBA1^+^ microglia at P2 and 80% at P7 [38]. Lastly, intranasally administered EL-4 (murine lymphoblast) EVs colocalized with more than 60% of IBA1^+^ cells within an hour of administration [44]. Ultimately, these studies reveal a specific uptake of Oli-Neu, SVZ, N2a, and EL-4 EVs by IBA1^+^ microglia in vivo*.*

#### *Functional effects of EVs on microglia* In Vivo

While a specific directed uptake of EVs from microglia has been established, one of the most important questions remains: what effect do EVs have on the polarization status of microglia in these non-diseased conditions? M1-like or M2-like polarization of microglia after EV interaction could substantially affect the pro- or anti- inflammatory status of the surrounding tissue. Microglia morphology and cytokine expression is correlated to microglia polarization. These three parameters were examined after EV uptake. In vivo experiments of Oli-Neu EVs revealed no change in morphology of EGFP-CX3CR1+ cells (a labeled chemokine receptor highly expressed in microglia) following EV colocalization. In vitro experiments revealed no increase in proinflammatory cytokines, such as TNF-α, IL-6, and IL-12, following Oli-Neu EV treatment of primary microglia [38]. This suggests Oli-Neu EVs do not have a significant effect on microglia polarization in non-diseased states. In contrast, in developmental models, microglia treatment with non-irradiated, DiI-labeled, SVZ NSC EVs did result in an increase of CD11b expression. A transition to reduced microglia complexity, quantified by number of extensions, compared to UV-treated EVs, suggested a more activated M1-like transition [43]. Therefore, the downstream effect of EVs on microglia polarization seems dependent on the EV-type applied.

Collectively, these studies show an important, specific relationship between CNS EVs and recipient microglia in developmental and non-diseased states summarized in Table 1. While the oligodendrocytes EVs were shown to have no effect on microglial morphology or cytokine release, SVZ NSC EVs were shown to invoke an amoeboid-like transition. To understand the overarching effects of stem cell or primary cell EV treatment on microglial polarization, an investigation of disease and injury models must also be considered.

**Table 1:** Interactions of EVs with microglia in homeostasis and development

*EV type*	*Recipient cell type*	*Functional effect or interaction*
Oligodendrocyte EVs (Oli-Neu EVs)	- Mixed primary and mono primary cultures C3cr1/ EGFP labeled	Robust and specific uptake [38, 41] PS-dependent specific uptake into microglia [38] No change in morphology or cytokine expression [38]
Neuroblastoma EVs (N2a EVs)	Primary microglia BV2 mouse microglial line	Specific uptake into microglia and not primary neuronal cells [39] Colocalization in vivo with 93% of IBA1 positive microglia at P2 and 80% at P7 [38]
Astrocyte derived EVs (ADEVs)	Primary microglia	Internalized by microglia and trafficked to microglial endosomes [40]. Decreased microglial phagocytosis following morphine – exposed ADEV treatment [40].
Subventricular zone neural stem cell- derived extracellular vesicles (SVZ NSC) EVs	In vivo microglia	Uptake into IBA1 and CD11b positive microglia [43]. Increase in CD11b expression and transition to rounded less complex cells [43].
Pheochromocytoma EVs (PC12 EVs)	MG6 mouse microglial line	Enhanced ability of microglia to remove degenerating neurite from PC12 cells due to upregulation of complement component 3 (C3). [42]. No effect on phagocytosis of E Coli [42].
Murine lymphoblast EVs (EL-4)	In vivo IB11^+^ microglia	Colocalization with greater than 60% of IBA1^+^ cells after intranasal administration [44]

**Fig. 1:**
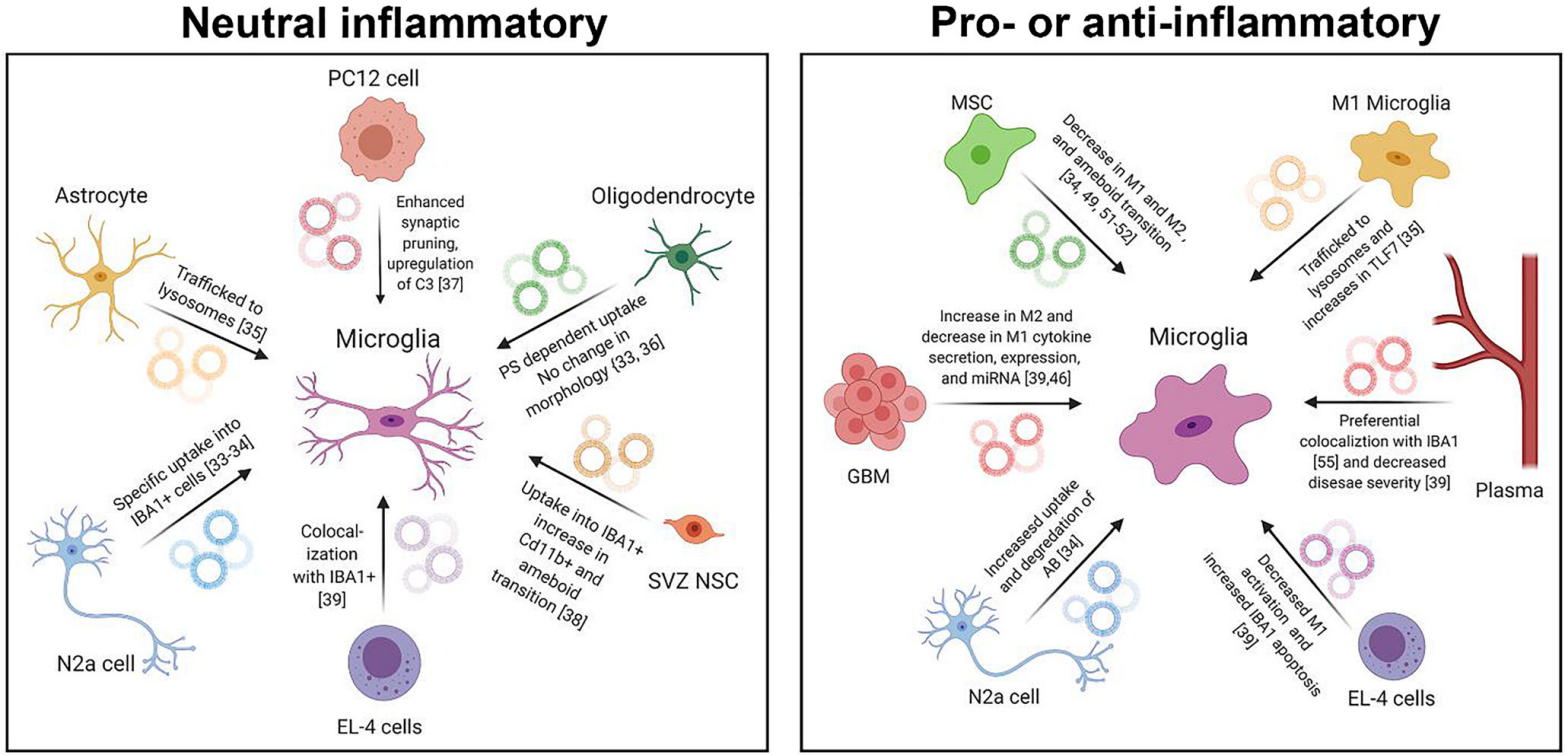
Diagram of effects of EVs on microglia in neutral (A) and pro- and anti- inflammatory states (B)

### The Role of EVs in Tumor Microenvironments

While EVs affect microglia in homeostatic states, their effect on the polarization of microglia in cancerous, inflammatory, and neurodegenerative conditions should also be considered, especially given the aforementioned therapeutic potential and interest in EVs. Historically, before reports of lymphatic vasculature along dural sinuses, the brain was considered an immune-privileged organ [45, 46]. Now, however, immune cells in the brain tumor microenvironment (TME) have emerged as a major regulator of tumor progression in low- and high-grade gliomas. Greater understanding of this TME can be utilized to understand tumor metastasis and tumor progression [47]. The interaction of tumor-derived EVs on immune cells in this environment should be considered.

#### Interaction of EVs and Microglia in Models of GBM

Glioblastoma multiforme (GBM), the highest-grade astrocytoma, is a heterogenous primary neoplasm highly resistant to current radiological, chemotherapeutic, and surgical options [48]. The therapeutic resistance of GBM results in only a 6% 5-year survival of this disease after diagnosis [49, 50]. In order to develop more targeted and effective treatment options, a better understanding of the intricate TME of GBM is warranted. A portion of the TME involving the interaction of GBM-derived EVs and microglia is discussed here.

Exposure of microglia to GBM EVs every 24 h for 5 days in vitro resulted in alterations in the secretion of multiple cytokines and a 40% increase in microglia proliferation. Out of 40 cytokines assessed, 6 cytokines were upregulated, and 3 cytokines were downregulated compared to non-exposed microglia by more than 50%. Of upregulated cytokines, 5 were involved in glioma growth, including CXCL10, CXCL1, CCL2, and CCL5 and IL-6, with 1, tissue inhibitor of metalloproteinase 1 (TIMP-1), being involved in matrix degradation. Of the 3 downregulated cytokines, IL-16, IL-23, and IL-27, all were involved in the induction of immune responses. Taken together, these results suggest that GBM EVs are able to significantly alter functional microglia phenotype, causing a progression from an M1 to a more M2-like phenotype evidenced through cytokine expression [51, 52]. This GBM EV invoked effect on dampening M1-like microglial activation could play a role in GBM immune evasion, as well as local immune dampening for metastasis priming [53]. Effects of GBM EVs on tumor-associated microglia is specific. Other cell populations, such as monocytes and macrophages, were not found to have significantly altered MT1-MMP expression following GBM EV treatment. Regional primary human microglia were, providing evidence of a microglia-specific response to GBM EVs compared to other immune cells [54]. Multiphoton intravital microscopy was used to confirm successful interaction of GBM EVs with microglia/monocytes in animals. GL261-FLuc-mcC-palmtdT tumors produced red-labeled EVs which could be tracked in their interaction with CX3CR1-GFP^+^ cells*.* Colocalization or interaction of EVs with microglia and monocytes was in 18–74% percent of tumor area, depending on field of view (FOV) location, as well as 1–10 puncta within GFP^+^ cells. Upon flow cytometry analysis there was a higher density of microglia associated with tumors and higher number of monocytes/macrophages overall in tumor-bearing tissue [51]. These studies show that GBM EVs do indeed interact with microglia.

#### Functional Effects of Loaded and Non-loaded EVs on GBM TME

To directly understand the downstream effects the GBM EVs are having on the polarization and function of recipient microglia, cytokine and phenotypic changes were studied. MiRNA transferred from GBM EVs to recipient microglia may invoke cytokine and phenotypic changes. Two miRNAs, miR-451 and miR-21 (out of 1146 assessed), were significantly elevated in patient-derived and immortalized GBM cells and EVs. Researchers assessed changes in these miRNAs in two recipient cell lines, primary mouse microglia and human adult primary microglia. Following addition of GBM EVs to primary mouse and human microglia, significant increases in miR-21 (a known oncomir expressed in solid tumors) and miR-451 (known to be associated with chemotherapy resistance) were observed compared to control, suggesting miRNA transfer from EVs to these cells. Additionally, *c-Myc* (a known target of both miR-21 and miR-451) mRNA levels were significantly decreased in recipient microglia following GBM EV treatment, suggesting functional miRNA transfer from GBM EVs to microglia. Finally, using FACS to examine and compare microglia from tumor-bearing mice to control mice, tumor-associated microglia had increased levels of miR-21 and decreased *c-Myc* mRNA levels, indicating functional miRNA transfer from EVs to microglia in vivo [51].

In addition to understanding the endogenous effects of GBM EVs on microglia and tumor progress, exogenous EVs can also be harnessed for therapeutic purposes in GBM. One approach is to load EVs with specific therapeutics destined for microglial targets. In an orthotopic glioblastoma (GL-26) model, intranasally administered EL-4 (murine lymphoblast) EVs loaded with JSI124 (STAT3 inhibitor) were shown to increase tumor apoptosis, survival, and concomitant reduction of tumor, as well as decrease neurological symptoms. Two animals treated with JSI124 EVs had no evidence of tumor left. Exo-JSI124 treatment was correlated with a reduction in STAT3 and decrease in IL-1β and IL-6 in CD45.2^+^ microglial cells, suggesting this beneficial treatment effect to be microglia-mediated. Ultimately, this study reveals the strength of EV-based therapeutics in the TME and specifically in GBM [44].

Collectively, these studies show that astrocytoma EVs are robustly taken up by recipient microglia in vivo and in vitro*, summarized in* Table 2*.* Of greater significance, this uptake is correlated with an increase in immunosuppression as well as conveyance of well-known onco-miRNA and chemotherapeutic resistance miRNAs. In light of these findings, inhibition of the uptake of GBM EVs or interruption to the downstream processing of GBM EV contents by microglia could prove a novel therapeutic avenue for GBM therapeutic development. Alternatively, exogenous EVs loaded with relevant inhibitors, such as JSI124, could effectively tune microglia responses to tumor cells, leading to decreased tumor burden and increased survival [44].

**Table 2:** Interactions of EVs with microglia in neural injury and disease

*Disease*	*EV type*	*Recipient cell type*	*Functional effect or interaction*
Glioblastoma Multiforme (GBM)	GBM EVs [51] [54] EL-4 EVs [44].	Primary mouse microglia KW3 Primary human GBM cells (11/5-) and (20/3) [51]	Increase in proliferation, expression of Arg-1mRRNA43], MT1-MMP [54] and CCL5, CCL2, CXCL1, CXCL10, TIMP-1 [51] Decrease in IL-27, IL-23, Il-17, and IL-16(51), STAT3, IL-1B, and Il-6 [44]. GBM EVs were seen interacting with CxCr1-GFP+ microglia in vivo [51] Increase of miRNA-21 and miRNA451 [51]
Spinal cord injury (SCI)	TNF-α and INF-γ stimulated mesenchymal stem cell EVs (MSCEV^+^) or non-stimulated MSC EVs (MSCEVs^wt^)	In vivo microglia	Decrease M1-like microglia (CD32^+^ and Cd86^+^) Decrease in M2-like microglia (Cd100R, Cd163, and RT1B) [56]
Perinatal brain injury through LPS injection at P3	MSC EVs	In vivo IBA1^+^ microglia	Decreased in number of IBA^+^ microglia and decreased ameboid transition [57]
Cortical injury in aged animals	-MSC EVs [58]	In vivo IBA1^+^ microglia	Increase in ramified MCHII expressing IBA1^+^ [58] Correlations between ramified morphology and functional recovery [58]
Alzheimer’s disease	N2a EVs exposed to Aβ [39]. Pre conditioned [65] MSC EV or MSC EVs [59].	BV2 microglia [39, 59]. Primary IBA1^+^ microglia [59].	Significant decrease in IBA1^+^ cells, TNF-a and IL-1B secretion and STAT3 and NF-kB expression [59]. Increases CD11c cells, IL-4, IL-10 secretion and mir-21[59]. Increased uptake, clearance, and degradation of Aβ by microglia in the presence of EVs [39;59].
Bacterial (LPS) challenge	Curcumin loaded EL-4 EVs (Exo-cur)	*-*In vivo Cd45.2^+^ and ILB^+^ microglia	Decrease in activated inflammatory microglia Increased microglial apoptosis [44].
Myelin oligodendrocyte glycoprotein (MOG)- induced experimental autoimmune encephalomyelitis (EAE)	Curcumin loaded EL-4 EVs (Exo-cur)	In vivo Cd45.2^+^ and ILB^+^ microglia	Decrease in activated inflammatory microglia, and decreased disease severity compared to curcumin alone and PBS [44].
Parkinson’s disease	Plasma derived EVs	In vivo IBA1^+^ microglia, and in vitro BV2 microglial	Colocalization with IBA1^+^ positive cells bilaterally, even though unilateral EV injection [63] Preferential internalization over neurons and astrocytes [63] Increase in IBA1^+^ cells and NO [63]
Traumatic brain injury (TBI)	Plasma derived EVs BV2 derived VEs	In vivo IBA1^+^ microglia, and in vitro BV2 microglial	Increases in IL-1B and CCL2 in BV2s with TBI primed plasma EVs Increases in IL-1B, TNF-a, CCL2, IL-6, AND NOS2 and ameboid transition in BV2 microglia with LPS primed BV2 EVs [64]
Opioid use	Human primary astrocyte derived EVs (ADEVs)	Mouse primary microglia	Increases in toll like receptor 7 (TLR7), NFkBp65 trafficking to the nucleus and decreases phagocytosis [40]

### EVs and Microglia in Neurodegenerative Disease

This anti-inflammatory effect of EVs could be extremely beneficial in neuroinflammatory or neurodegenerative diseases. While the effects of microglia-*derived* EVs in a range of neurodegenerative diseases have been examined in recent reviews [36, 37, 55], the effects of CNS and non-CNS EVs *on* microglia in these diseases should also be coalesced. Here, the effects of EVs from various cell types in different CNS diseases and injuries is discussed in in vitro and in vivo models.

#### Interaction of EVs and Microglia in Neuroinflammatory Injury and Disease

In a model of spinal cord injury (SCI), treatment with EVs from TNF-α- and IFN-γ-stimulated mesenchymal stem cells (MSC EVs^+^) or EVs from non-stimulated mesenchymal stem cells (MSC EVs^wt^) at 3 h post-injury was able to significantly decrease M1-like microglia (CD32^+^ and CD86^+^) levels to those of sham animals. Concentration of M2-like microglia (CD100R^+^, CD163^+^, and RT1B^+^) was also decreased with MSC EVs^+^ and MSC EVs^wt^ treatment, revealing an overall attenuation of microglial responses by MSC EVs following SCI [56]. The anti-inflammatory effects of MSC EVs on microglia in other disease models has been shown, as well. In a model of perinatal brain injury, MSC EVs decreased the number of IBA1^+^ cells after systemic LPS injection at P3, compared to LPS stimulation alone. Amoeboid transition, associated with M1-like proinflammatory microglia, was also decreased with MSC EV treatment [57]. This shows that MSC EVs induced a transition of microglia away from M1 polarization in two different models of acute injury. In an aged rhesus monkey model of cortical injury, EV treatment resulted in a greater density of ramified MHCII-expressing microglia. Morphological features of microglia after EV treatment were significantly correlated with motor function recovery in these animals [58]. MSC EVs were able to effectively attenuate microglia activation even in a perinatal injury model. This was in contrast to earlier developmental studies discussed, which showed an increase in M1 polarization of microglia following interaction with SVZ NSC EVs. This implies that EV effects on microglial polarization may be more specific to EV source and overarching inflammatory state than developmental timepoint.

MSC EVs were also found to be an effective anti-inflammatory therapeutic in a mouse model of Alzheimer’s disease (AD). Transgenic AD mice were treated with MSC EVs or pre-conditioned MSC EVs (PC-MSC EVs). DiI-labeled EVs from both treatment groups were shown to colocalize with astrocytes and microglia upon imaging. Additionally, the expression of IBA1 in microglia was significantly decreased in both EV-treated groups, and there was a significant increase in CD11c^+^ cells (pan-myeloid marker) in the PC-MSC EVs treatment group. Furthermore, analysis of cytokine secretion revealed PC-MSC EV and MSC EV treatment resulted in a decrease of TNF-α and IL-1β cytokine secretion and gene expression and increased the levels of IL-4 and IL-10 (anti-inflammatory cytokines). Additional in vitro experiments confirmed a significant reduction in TNF-α and IL-1β of LPS-treated BV2s after PC-MSC EV or MSC EV treatment. Lastly, a decrease in STAT3 and NF-κB p65 (inflammatory pathways) activation was observed in the brains of AD mice, and an increase in miR-21 expression was detected after PC-MSC EV or MSC EV treatment. In each of these measures, PC-MSC EVs showed even more significant anti-inflammatory effects than non-pre-conditioned MSC EVs. In these studies, authors speculate that these anti-inflammatory effects of MSC EV treatment could be responsible for drastic improvements in learning seen in AD mice during Morris water maze assessment [59]. Collectively, these studies of neuroinflammatory conditions reveal an overarching anti-inflammatory effect of MSC EV treatment, either with or without pre-treatment, on microglia polarization.

Microglia are known to play an important role in amyloid beta (Aβ) degradation in models of AD. A role for EVs in Aβ trafficking and degradation was investigated. Aβ-exposed to N2a EVs was taken up more readily by BV2s and primary microglia and was cleared faster from the media. This increased clearance and uptake of Aβ by microglia was dependent on PS availability on N2a EVs. Once internalized, Aβ-laden EVs were trafficked to the lysosomes of recipient microglia for degradation [39]. Also, Alzheimer’s disease mice were treated with MSC EVs or PC-MSC EVs (mentioned above). There was a reduction in plaque deposition in the cortex and hippocampus of transgenic AD mice treated with MSC EVs or PC-MSC EVs. Most compelling, there were significant decreases in soluble and insoluble Aβ observed in both EV-treated groups, implying MSC EV treatment can also halt the spread of AD to other locations within the brain [59]. These studies reveal how this aforementioned relationship between EVs and microglia is utilized for clearance and degradation of pathological materials in the diseased brain [39].

Like Alzheimer’s disease, Parkinson’s disease [60] remains a leading cause of neurodegeneration and chronic neuroinflammation [61]. In 6-hydroxydopamine-invoked models of PD, measured increases in TNF-α, IFN-γ, IL-1β, IL-2, IL-6, and a decrease in IL-10 indicate the role microglia play in perpetuating this proinflammatory condition [62]. Peripheral immune cells may also be contributing to this increased proinflammatory state, in turn further perpetuating microglial activation. To answer this question, researchers isolated EVs from the plasma of PD patients and analyzed their interactions with mouse microglia in vitro and in vivo. Following application to BV2s, plasma EVs were shown to be taken up, colocalize with microglia lysosomes, and activate the Akt-mTOR pathway, suppressing autophagy, a crucial process in α-synuclein (α-syn) degradation. Upon unilateral injection of PKH26-labeled PD patient plasma EVs, specific colocalization with IBA1^+^ cells was observed in the bilateral striatum, substantia nigra, and cortex, and an increase in IBA1 and NO expression and a transition to amoeboid microglia phenotype was observed. Increased phosphorylated α-syn accumulation in dopaminergic neurons of the substantia nigra pars compacta (SNpc) lends to a hypothesis of EV-mediated microglia-to-neuron mode of transmission for α-syn conformers [63]. This study reveals that endogenous EVs, exposed to the already proinflammatory PD environment, interact with microglia, further perpetuating neuroinflammation and potentially the spread of α-syn.

The downstream effects of proinflammatory-exposed blood-derived EVs were also considered in a mouse model of TBI. Flow cytometry analysis revealed that microglia-derived EVs make up a significant portion of the total EV population and were also increased after TBI. Exposure of BV2 cells to enriched blood EVs from TBI animals resulted in significant increases in IL-1β and CCL2 in recipient microglia and no significant change in TNF-α or miR-155 compared to non-TBI-exposed EVs [64]. LPS-exposed BV2 EVs also resulted in increases in IL-1β, TNF-α, CCL2, IL-6, NOS2 mRNA, and miR-155 in recipient BV2 microglia. In addition, LPS-exposed BV2 EV treatment resulted in significant increases in IBA1^+^ and P2Y12^+^ cells and an amoeboid transition in in vivo microglia [64]. Taken together, these TBI and PD examples reveal that plasma EVs which are previously exposed to proinflammatory conditions perpetuate further proinflammatory responses in recipient microglia. EVs derived from cells which have not been previously stimulated, however, show a net decrease in proinflammatory activation of recipient microglia, summarized in Table 2.

#### Loading of EVs with Anti-Inflammatory Therapeutics for Targeted Microglial Delivery

As in previously mentioned GBM experiments, EVs can be loaded with certain molecules to increase delivery of intended therapeutics to microglia due to this specific targeted uptake. In neuroinflammatory conditions, anti-inflammatory therapeutics can be loaded into EVs for targeted delivery to microglia. In one study, administered curcumin (spice known to suppress inflammation)-loaded EL-4 (mouse lymphoma cell line) EVs (Exo-cur), were shown to significantly reduce the number of activated inflammatory microglia (CD45.2^+^ IL-1β^+^) after LPS challenge compared to non-treated animals. Furthermore, apoptosis was confirmed in microglia cells through TUNEL assay, revealing efficacy of loaded EVs not only to decrease microglia activation and proliferation, but also to induce cell death [44]. In a myelin oligodendrocyte glycoprotein (MOG)-induced experimental autoimmune encephalomyelitis (EAE) model, disease severity in Exo-cur mice was significantly reduced compared to vehicle and non-encapsulated curcumin-treated mice. This significant functional effect of Exo-cur EVs was correlated with decreased expression of IL-1β and CD45.2^+^ microglia [44]. The significant differences observed between Exo-cur and non-encapsulated curcumin groups provides support for the use of EVs for targeted therapeutic delivery.

## Concluding Remarks

This review was conducted to coalesce the current understanding of the effects of EVs on microglia in development, homeostasis, the TME, and in neurodegenerative disease. While EVs from some sources, such as SVZ NSCs, did show a polarization of microglia to a more M1-like morphology, CNS EVs from all other studies presented here showed measurable anti-inflammatory effects (in disease states) or no effects (in non-diseased states), evidenced by cytokine release, expression, and morphological analysis. It should be noted that many of EVs referenced in this review were derived from immortalized or cancerous cell lines which are not found in normal CNS microenvironment. Therefore, the in vivo microenvironment and microglial response may differ. Even with this caveat, these findings suggest the relationship between EVs and microglia is very specific, measurable, and of therapeutic interest.

In the TME, pre-clinical studies have already begun to assess the efficacy of increasing M1-like activation of microglia in an effort to decrease tumor burden. Specifically, differentiation of M2-like microglia to a more M1-like phenotype through competitive antagonism of the colony stimulating factor-1 receptor (CSF-1R) was shown to halt GBM growth and enhance survival [66]. Therefore, the efficacy of shifting microglia polarization to enhance survival in GBM has been established clinically. Inhibition and/or sequestration of endogenous GBM EVs or loading of exogenous EVs with M2 inhibitors (which will be specifically taken up by microglia) have the potential for similar efficacious outcomes through halting the characteristic M2-like polarization of GBM-associated microglia.

In cases of disease or trauma-invoked neuroinflammation, inhibition of microglial activation has been an efficacious strategy. For example, corticosteroids have been shown to inhibit microglial activation in SCI [48] and traumatic brain injury (TBI) [67], and minocycline-induced attenuation of M1 microglial responses has shown efficacy in stroke [68] and Parkinson’s disease [69]. Attaining this level of anti-inflammation has been a goal of many regenerative medicine whole cell biological strategies, such as MSCs [70]. There exists, however, many limitations of whole cell therapies, including risk of rejection, site accessibility, and retention [12, 13, 71–73). EV-based therapeutics exist in a new space between the two approaches, with enhanced multi-faceted therapeutic potential over single-target small molecule strategies and without some of the limitations associated with whole cell therapies. Through exogenous administration of EVs or enhancement of endogenous EV production, anti-inflammatory efficacy through targeting of microglia polarization in various neuronal diseases could be achieved.

## Abbreviations

AD: Alzheimer’s disease
ADEVs: astrocyte derived extracellular vesicles
CSF1: colony stimulating factor 1
CSF-1R: colony stimulating factor-1 receptor
C3: complement component 3
Exo-cur: curcumin-loaded mouse lymphoma cell EVs
CINC: cytokine-induced neutrophil chemoattractant
eGFP: enhanced green fluorescent protein
EAE: experimental autoimmune encephalomyelitis
EVs: Extracellular vesicles
FOV: field of view
GBM: glioblastoma multiforme
IBA: ionizing calcium adaptor binding protein
JS1124: STAT3 inhibitor
BV2: microglial cell line (BV2)
MG6: mouse microglia cell line
EL-4: murine lymphoblast
MOG: myelin oligodendrocyte glycoprotein
NSC: neural stem cell
N2a: neuroblastoma cell line
NC: neurosphere cells
MSC EVs^wt^: non-stimulate mesenchymal stem cell
Oli-Neu: oligodendroglia cell
GL-2: orthotopic glioblastoma
60: Parkinson’s Disease
PC12: pheochromocytoma
73: phosphatidyl choline
PS: phosphatidyl serine
PC-MSC EVs: pre-conditioned MSC EVs
SCI: spinal cord injury
MSC EVs^+^: stimulated mesenchymal stem cells
SNpc: substania nigra pars compacta
SVZ: subventricular zone
NIH3T3/ TIM4: TIM4 expressing mouse embryonic fibroblast line
TIMP-1: tissue inhibitor of metalloproteinase 1
TLR7: toll-like receptor 7
TGF-β: transforming growth factor beta
TBI: traumatic brain injury
TME: tumor microenvironment
YS: yolk sac
α-syn: α-synuclein
